# A unique case of bilateral hydronephrosis caused by a fecalith

**DOI:** 10.1002/ccr3.1203

**Published:** 2017-11-23

**Authors:** Archana Kulkarni, Maryann Kimoto, Rafael Morales, Amit Kaura

**Affiliations:** ^1^ Department of Internal Medicine Allegheny Health Network Pittsburgh Pennsylvania; ^2^ Department of Critical Care and Pulmonology Allegheny Health Network Pittsburgh Pennsylvania

**Keywords:** Constipation, fecalith, hydronephrosis, polyethylene glycol electrolyte solution

## Abstract

This is a unique case that signifies the importance to look beyond the genitourinary system for causes of hydronephrosis. In addition, we outline the manner in which a fecalith should be addressed.

## Case Report

**Figure 1:**
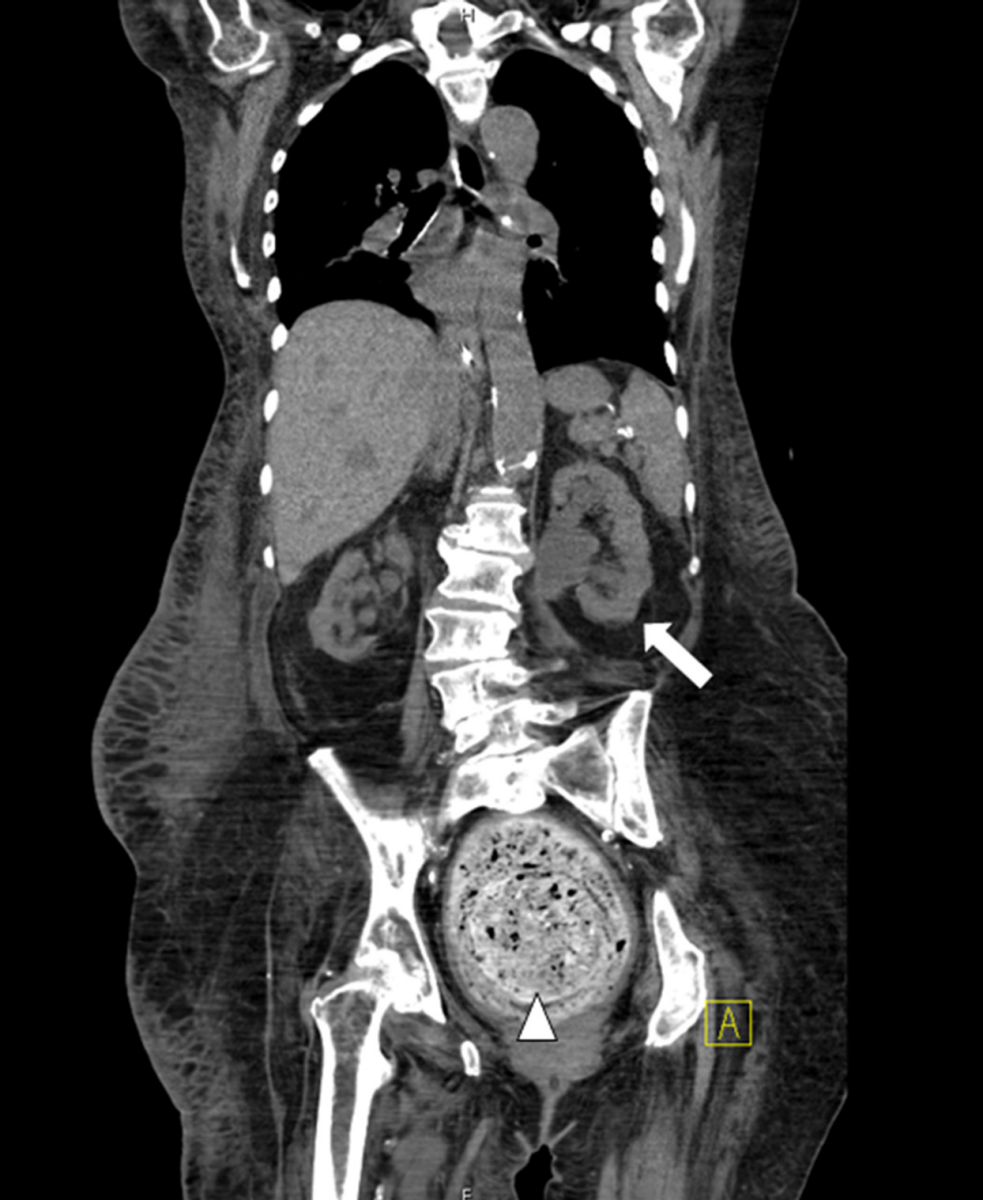
Noncontrast CT abdomen/pelvis, coronal view. Rectal fecalith (14 cm; triangular arrow) with subsequent compression of the ureters and resulting bilateral hydronephrosis and hydroureter (white arrow).

**Figure 2:**
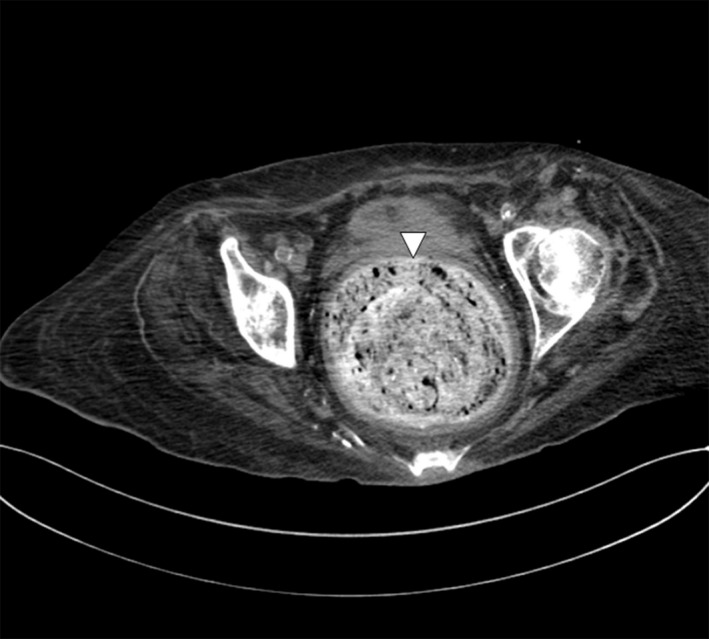
Transverse view of large, rectal fecalith (triangular arrow).

**Figure 3:**
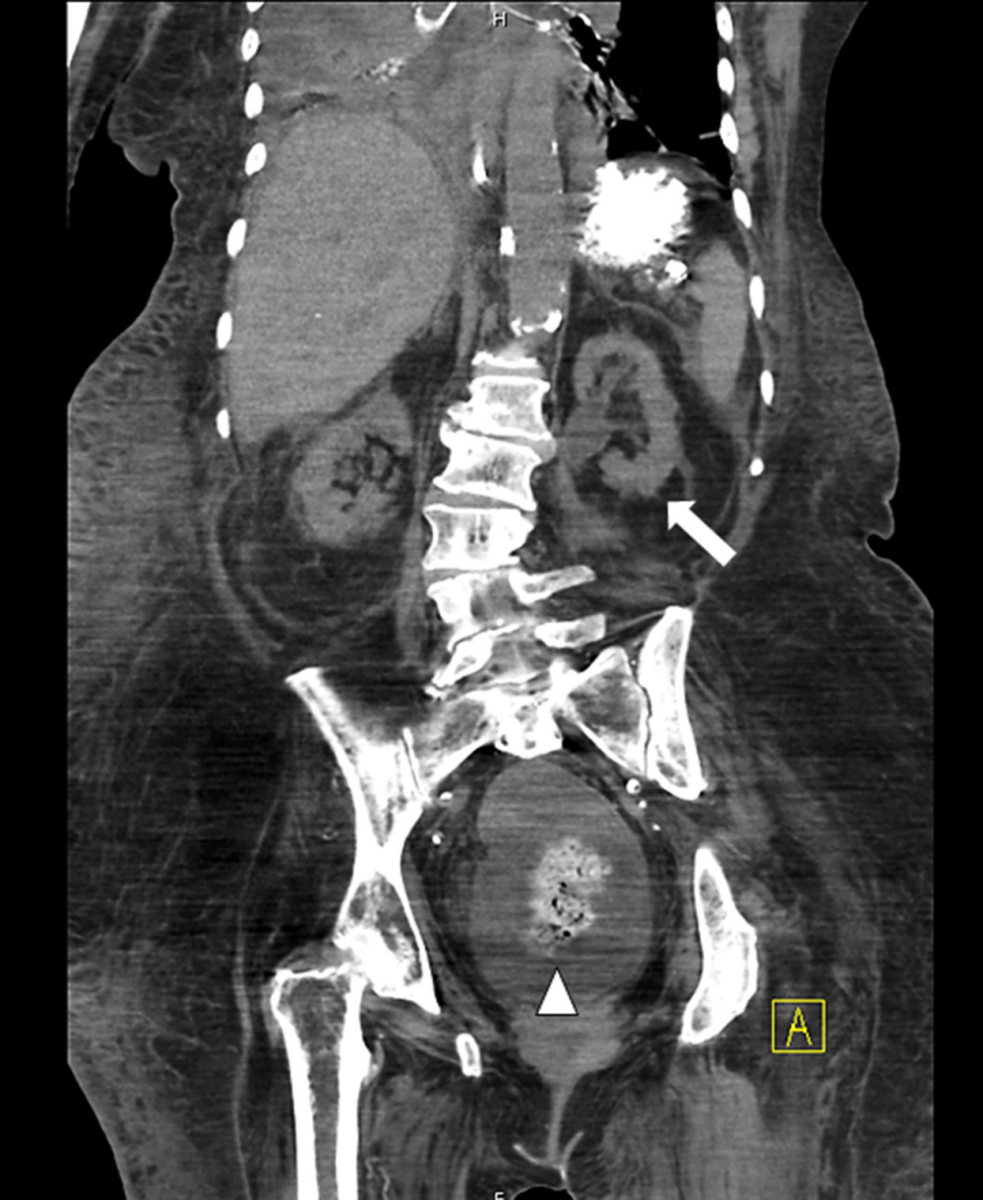
Noncontrast CT abdomen/pelvis, coronal view. Postdisimpaction and aggressive medical management. There is interval decrease in size of the rectal fecalith to 4 cm (white arrow) with persistent hydronephrosis and hydroureter (triangular arrow).

**Figure 4:**
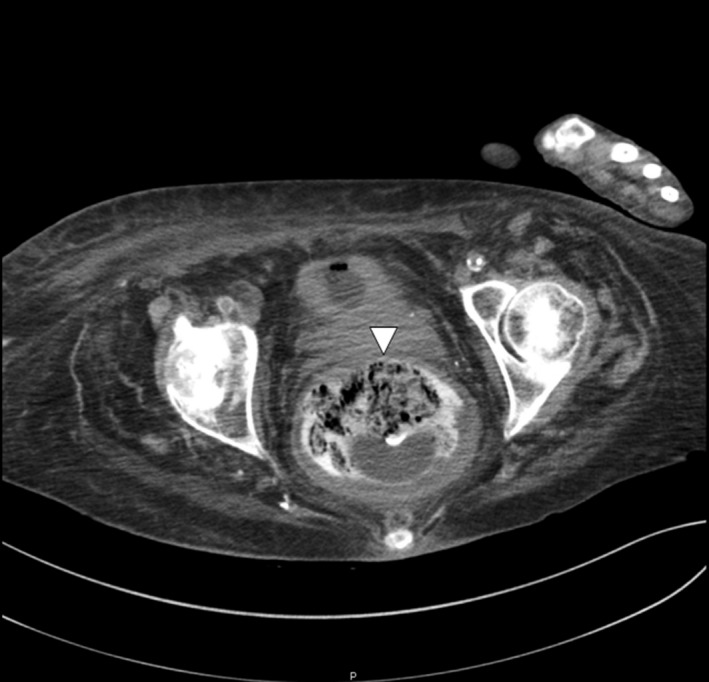
Transverse view of resolving rectal fecalith (triangular arrow).

We report a case of a rectal fecalith resulting in bilateral hydronephrosis and hydroureter. A 76‐year‐old woman presented with minimal urine output and generalized weakness. She was diagnosed with septic shock secondary to a urinary source. Laboratory studies were consistent with acute renal failure (4.0 mg/dL on presentation‐baseline creatinine 0.7) and uremia (BUN 108 mg/dL). A noncontrast computed tomography (CT) scan of her pelvis showed bilateral hydronephrosis and hydroureter secondary to a large (14 cm) rectal fecalith (Figs. 1 and 2). Manual disimpaction was performed, and an aggressive bowel regimen of fiber laxatives, stool softeners, and enemas were given. Large amounts of stool were fragmented. A polyethylene glycol electrolyte solution at a rate of 100 mL/h for 24 h was administered for fecalith resolution. A repeat CT scan of her abdomen and pelvis on hospital day 2, revealed a decrease in size of the fecalith to approximately 4 cm. Persistent bilateral hydronephrosis and hydroureter were observed; however, slow resolution postdisimpaction was an expected outcome (Figs. 3 and 4). While the patient's creatinine trended down to 1.53 mg/dL by hospital day 9, her hospital course was complicated by aspiration pneumonia, and she was discharged to hospice care.

## Consent Confirmation

Consent was obtained from the patient for publication of case details.

## Conflict of Interest

None declared.

## Authorship

AK: Contributed to write the case and identify the images. MK: Contributed to write the case and identify the images. RM: Reviewed and edited the case report and helped in identifying appropriate images. AK: Reviewed and edited the case report and helped in identifying appropriate images.

